# On the correlation between solar activity and large earthquakes worldwide

**DOI:** 10.1038/s41598-020-67860-3

**Published:** 2020-07-13

**Authors:** Vito Marchitelli, Paolo Harabaglia, Claudia Troise, Giuseppe De Natale

**Affiliations:** 1 Scuola di Ingegneria, Università della Basilicata, Via dell’Ateneo Lucano 10, 85100 Potenza, Italy; 2 Department of Mobility, Public Works, Ecology, Env., Puglia Region Government, 70100 Bari, Italy; 3 Istituto Nazionale di Geofisica e Vulcanologia, Via Diocleziano 328, 80124 Naples, Italy; 4 CNR-INO, Via Campi Flegrei 34, 80078 Pozzuoli, Italy

**Keywords:** Seismology, Solar physics

## Abstract

Large earthquakes occurring worldwide have long been recognized to be non Poisson distributed, so involving some large scale correlation mechanism, which could be internal or external to the Earth. Till now, no statistically significant correlation of the global seismicity with one of the possible mechanisms has been demonstrated yet. In this paper, we analyze 20 years of proton density and velocity data, as recorded by the SOHO satellite, and the worldwide seismicity in the corresponding period, as reported by the ISC-GEM catalogue. We found clear correlation between proton density and the occurrence of large earthquakes (*M* > 5.6), with a time shift of one day. The significance of such correlation is very high, with probability to be wrong lower than 10^–5^. The correlation increases with the magnitude threshold of the seismic catalogue. A tentative model explaining such a correlation is also proposed, in terms of the reverse piezoelectric effect induced by the applied electric field related to the proton density. This result opens new perspectives in seismological interpretations, as well as in earthquake forecast.

## Introduction

Worldwide seismicity does not follow a Poisson distribution^1^, not even locally^2^. Many authors have proposed specific statistical distributions to describe such a non-poissonian behavior^3–7^ but none of these is really satisfactory, probably because the underlying physical process has not been really understood. Many authors have hypothesized that a tidal component may show up in earthquake activity (e.g.^8,9^) but generalized evidence has never been proven. Quite recently, some authors^10^ suggested that earthquake occurrence might be linked to earth rotation speed variations. There is also a smaller number of researchers that studied possible links among solar activity, electro-magnetic storms and earthquakes (e.g.^11–16^). The first idea that sunspots could influence the earthquake occurrence dates back 1853, and is due to the great solar astronomer Wolf^17^. Since then, a number of scientists has reported some kind of relationship between solar activity and earthquake occurrence^16,18,19^; or among global seismicity and geomagnetic variation^15,20^ or magnetic storms^21,22^. Also, some mechanisms have been proposed to justify such correlations: small changes induced by Sun-Earth coupling in the Earth’s rotation speed^23^; eddy electric currents induced in faults, heating them and reducing shear strength^24^; or piezoelectric increase in fault stress caused by induced currents^25^. However, none of these studies allowed achieving a statistically significant conclusion about the likelihood of such mechanisms. On the contrary, ^26^ argued that there is no convincing argument, statistically grounded, demonstrating solar-terrestrial interaction favoring earthquake occurrence. However, the large interest nowadays for possible interactions between earthquake occurrence and extra-terrestrial (mainly solar) activity, is testified for instance by the Project CSES-LIMADOU, a Chinese–Italian cooperation aimed to launch a satellite to study from space the possible influence of solar activity and ionospheric modifications on the seismicity^27^. In this paper, we will definitively establish the existence of a correlation between solar activity and global seismicity, using a long data set and rigorous statistical analysis. Once such a correlation is demonstrated, we propose a tentative, at the moment qualitative, mechanism of possible sun–earthquakes interaction.

### Statistical assessment of the correlation solar activity–earthquakes

Since our aim was to verify the existence of a link between solar activity and earthquakes, we considered two data sets: worldwide earthquakes, and SOHO satellite proton measurements.

As far as earthquakes are concerned, we used the ISC-GEM catalogue^28^. We choose it since, at the moment, this is the only worldwide data set with homogeneous magnitude estimates that allows for sound statistical analysis. We selected this catalog since it is the only complete one, with homogeneous magnitudes, albeit only from M = 5.6. It in fact includes all the Global CMT solutions^29,30^ and adds about 10% of events that are missed by the latter. In this cases magnitudes are expressed as mb and Ms proxies. We checked its completeness for *M* ≥ 5.6 since 1996. The earthquake catalogue currently (*ver.* 7.0) goes up to the end of 2016. The earthquake catalogues we used throughout this paper, with progressively larger magnitude threshold, are reported in Table 1.

**Table 1:** Earthquake data sets used in this paper.

Start time	1996-01-21
End time	2016-12-31
SOHO available days	6,472
Minimum magnitude	Events occurred in SOHO Available Days
5.6	6,612 (Total catalogue)
5.6	2,937 (first half-catalogue, ‘learning’)
5.6	3,675 (second half-catalogue, ‘testing’)
5.6	5,290 (Shallow catalogue: D < 60 km)
5.6	1,322 (Deep catalogue: D > 60 km)
6.0	2,491
6.5	789
7.0	250
7.5	93
8.0	18

Events are extracted from the ISC-GEM catalogue.

The SOHO (Solar and Heliospheric Observatory) satellite is located at the *L*1 Lagrange point at about 1.5 millions of kilometers from the Earth. Hourly data in terms of proton density *ρ* and velocity *v* are available for about 85% of the time since early 1996. Combining the two variables in the catalogue, we could infer, as further variables, the proton flux *ρv*, and the dynamic pressure *ρv*^2^/2. We have therefore considered, in our analyses, four different proton variables *V*: flux, dynamic pressure, velocity, and density. We computed the average of each proton variable in consecutive daily intervals. In Table 2 we report minimum, maximum, and average values for each variable *V*.

**Table 2:** The four variables *V* we used in this paper.

Variables	Min. daily av	Av. daily av	Max. daily av
Prot. density ρ (cm^−3^)	0.26	5.64	40.33
Prot. velocity v (km s^−1^)	270	425	957
Prot. flux ρv (cm^−3^ km s^−1^)	147	2,297	16,492
Prot. Dyn. Press. ρv^2^/2 (cm^−3 ^km^2^ s^−2^)	30,135	492,185	4,965,809

Proton density *ρ* and velocity *v* are from CELIAS/PM experiment on the Solar Heliospheric Observatory (SOHO). The other two are derived.

As a first step, each one of these variables *V* has been compared with the worldwide seismic events with *M* ≥ 5.6 in the period 1996/01/21-2016/12/31, considering the daily number of events only. The choice of this data set is due to the fact that it is the largest one. The daily number of events is more significant than the daily total moment, since we are interested in the number of individual rupture processes, rather than in a quantity that spans several orders of magnitude. Moreover, for large numbers of events, over a few thousands, the Gutenberg Richter relation^31^ is universally valid and, since earthquakes are self-similar, the number of events equivalently reflects the size of the main shock. We also chose not to decluster the event data set for two reasons. First, according to^32^, it is wrong to distinguish between main events, aftershocks, and background activity; second, declustering is somewhat arbitrary but would anyway result in a completely uncorrelated catalogue, likely destroying key information we are looking for.

Proton density and velocity vary with time, so if any correlation with earthquakes does exist, it must be found either in terms of different earthquake rates according to high/low proton values, or before/after the high or low values. We hence decided to investigate 5 conditions that are illustrated in Table 3.

**Table 3:** The 5 conditions examined to verify an eventual correlation with earthquakes, given a threshold *V*_*T*_ for any of the variables *V*.

Label	Description
aT	All the days with *V* above the *V*_*T*_ threshold
2lstDy aT	2nd to last day with *V* above the *V*_*T*_ threshold
lstDy aT	Last day with *V* above the *V*_*T*_ threshold
1Dy bT	1st day with *V* below the *V*_*T*_ threshold
2Dy bT	2nd day with *V* below the *V*_*T*_ threshold

**Figure 1:**
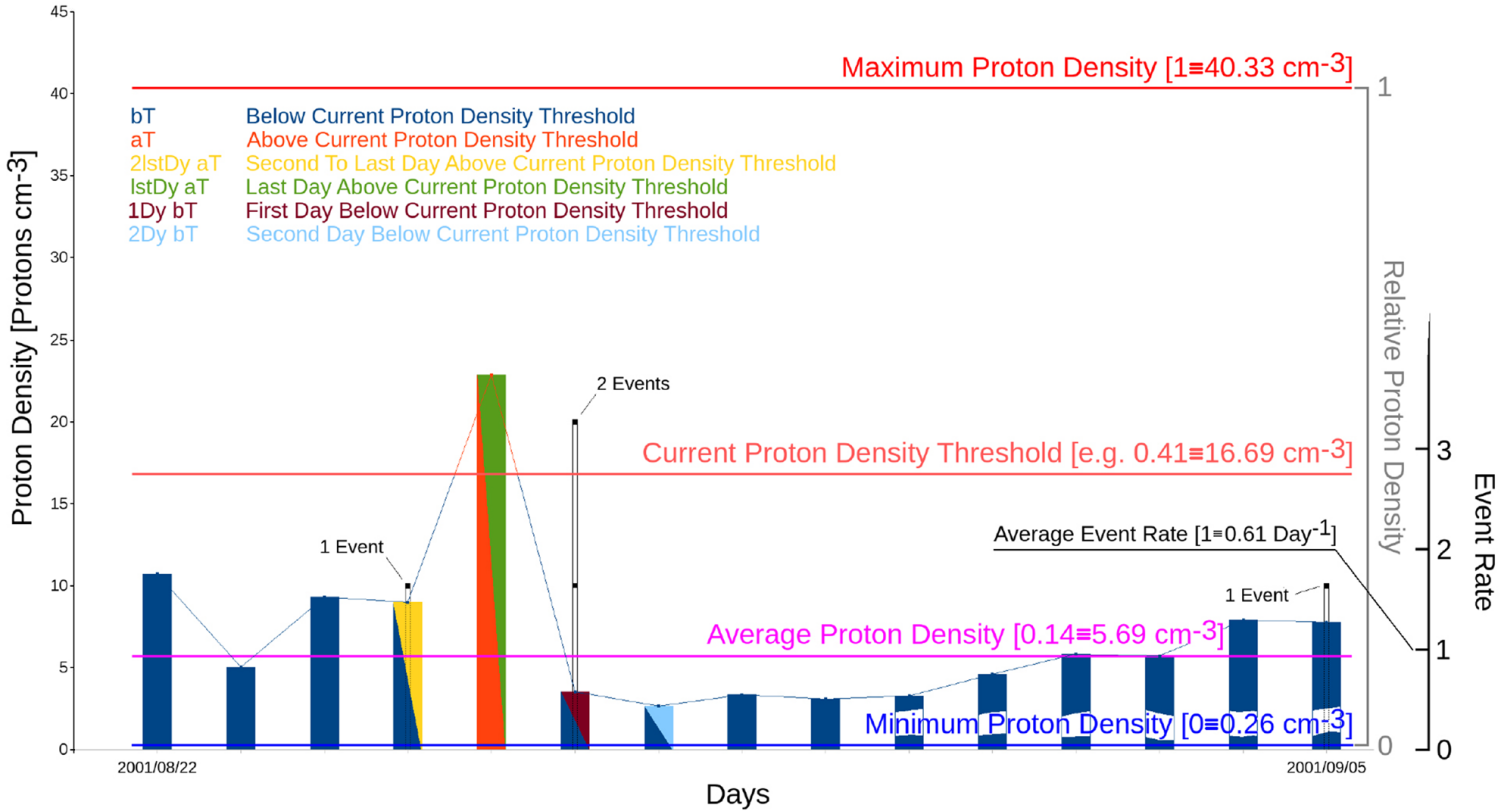
The figure shows an example of application of the statistical method to 15 days of the catalogue. The istogram levels give the daily value of the proton density; the red line shows the level of the current density threshold (all values of it are consecutively tested). The black points indicate the occurrence of earthquakes in that day. The istogram colours indicate the conditions which are applied for the statistical tests; in particular, violet indicates the first day below the current proton density threshold (i.e. the first day after a value above the threshold), the green indicates the last day above the density threshold, and so on (as indicated in the legend). High values of earthquake frequency in one of these particular periods indicate the tendency of earthquakes to occur before, during, after (and with what time lag) a period of proton density above the current threshold. Also shown in the figure are the minimum (blue), average (purple) and maximum (intense red) values of proton density for the whole catalogue used.

Figure 1 shows, with an example made on 15 days of catalogue, the overall procedure and illustrates the meaning of the used conditions for the statistical tests.

Another important remark is that since we consider 4 variables, 5 conditions and, later in the discussion, 6 magnitude thresholds with different temporal windows, we choose to use non-dimensional algorithms, to facilitate comparison.

The first step consists in computing the average of *V *(*V*_*av*_)*.* Because of the necessity of working with non-dimensional variables, we express the non-dimensional average of *V* (*V*_*av_ad*_) as

1 $$V_{{av - ad}} = (V_{{av}} - V_{{\min }}) /(V_{{\max }} - V_{{\min }})$$

approximated to the second significant digit. Then, we define a varying threshold, as

2 $$V_{T} = \, V_{min} + \, V_{step} \left( {V_{max} - V_{min} } \right)$$

for each variable *V*, where *V*_*step*_ ranges from the average value of *V*_*av_ad*_ to 1, with steps of 0.01. For a given condition C, and for each *V*_*T*_*,* we can count the number *D*_*C*_ of days that satisfies the condition and the corresponding number of events *E*_*C*_ occurring in those days. *D* and *E* are respectively the number of days where SOHO data are available and the total number of events that occur in those days. In this way for each *V*_*T*_, we can simply define an event relative rate

3 $$R = \left( {E_{C} /D_{C} } \right)/\left[ {\left( {E - E_{C} } \right)/\left( {D - D_{C} } \right)} \right]$$

**Figure 2:**
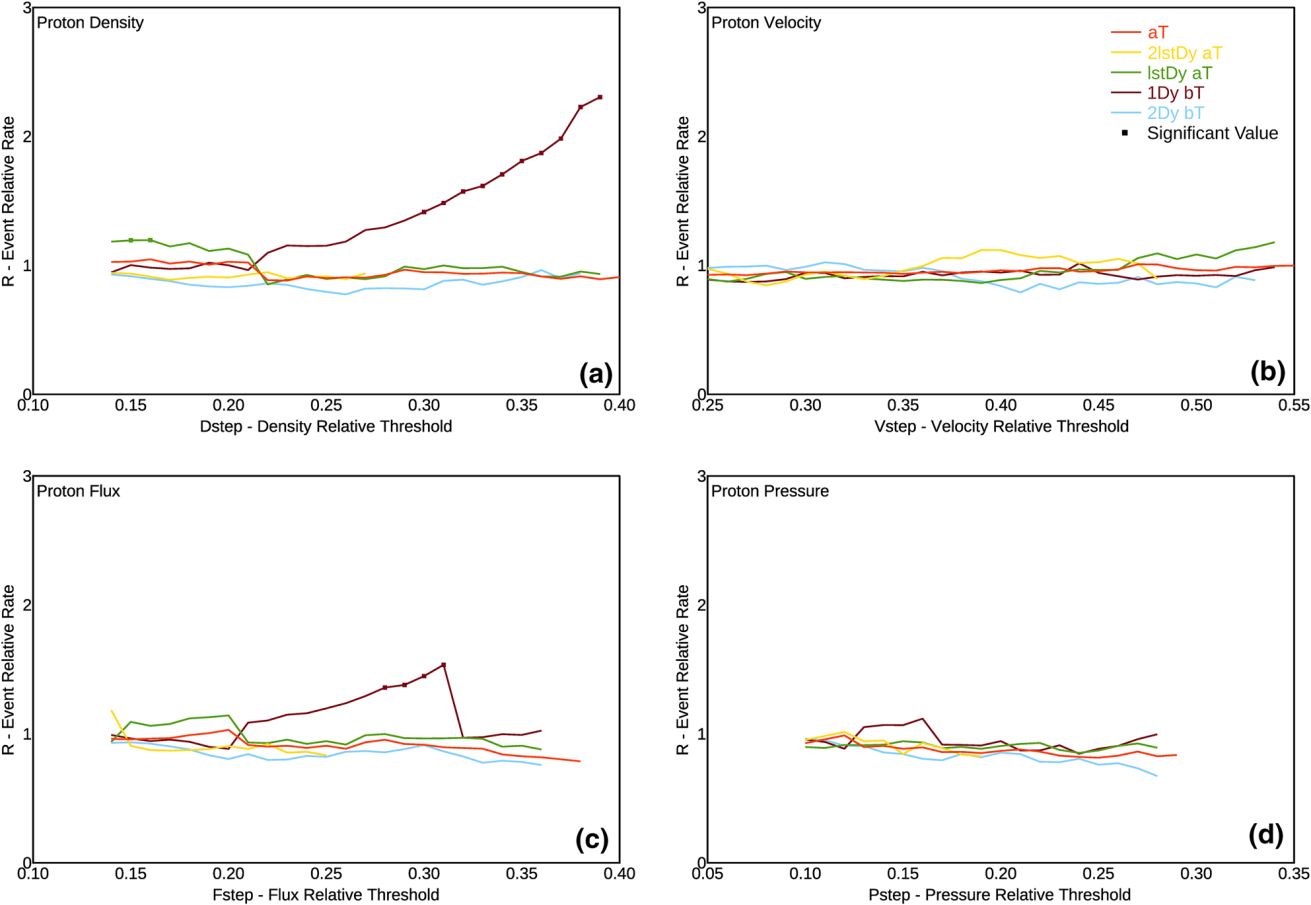
Plots of the Event Relative Rate (see Eq. 2) as a function of the non-dimensional Density Threshold, for: (**a**) proton flux; (**b**) proton dynamic pressure; (**c**) proton velocity; (**d**) proton density. Different colours refer to different conditions, as explained in Table 2. Squares refer to values which show statistically significant correlation at a significance level 0.00001.

In Fig. 2 we show the event relative rate *R* versus *V*_*step*_, for each condition C, represented for the 4 variables: flux, dynamic pressure, velocity, and density. This approach implies that, if earthquakes occur casually with respect to proton variables *V*, the event relative rate *R* should oscillate around 1, within a random uncertainty.

For most of the *CV* pairs shown in Fig. 2, we stopped computation at *V*_*step *_*≈ *0.4. This is due to the fact that, for larger threshold values, *D*_*C*_/*D* becomes smaller than 0.015, thus giving a too poor sampling. This value has been selected so to have at least about 100 days satisfying the selected condition.

**Figure 3:**
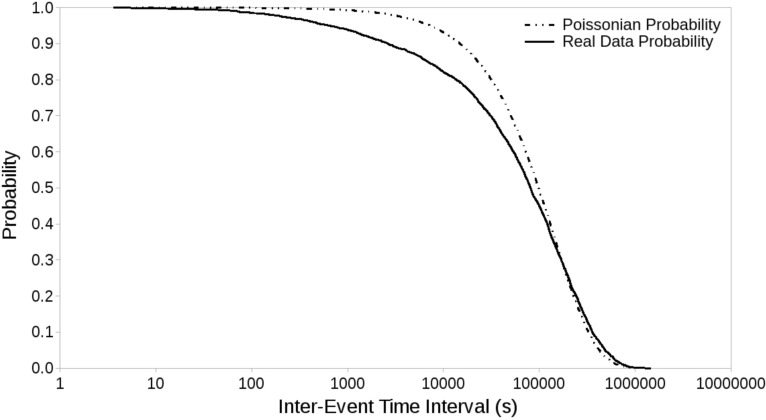
Inter-arrival time distribution of the events in the seismic catalogue (solid line). The dotted line shows, for comparison, the expected distribution of inter-arrival times for a Poisson distribution with the same event rate.

The final step consists in evaluating if *R* is significantly different from 1, for any of the variables *V,* in any of the conditions *C* within a *V*_*T*_ range. This means we need to devise a test starting from the assumption that earthquake occurrence is not poissonian^1–7^. We choose to create 10^5^ synthetic data sets, using the real data inter-event time intervals randomly combined. This empirical approach ensures us a synthetic catalog that has exactly the same statistical properties as the actual one, since we obtain a random data set with the same survival function as the real one. The survival function gives the probability of occurrence of inter-event time intervals and is commonly used to describe the statistical properties of earthquake occurrence (e.g.^1,4^). We followed this empirical approach because, as stated above, there is no satisfactory distribution that describes inter-event time intervals in a non declustered event series. To clarify our approach, in Fig. 3 we compare the real event survival function with a Poissonian one with identical event rate. As it is clear, the inter-arrival times of the real catalogue are markedly different from a Poisson distribution.

We wanted therefore to test whenever any random distribution could casually yield the same effects, in terms of *R* values, as the real one. Only if, for a given *V*_*T*_*, R* is higher than any of the values *R*_*rand*_ obtained by randomly distributed time intervals distributions, we consider that value as significant, thus clearly indicating correlation. This bootstrap technique corresponds to perform a statistical test with the null hypothesis that the observed correlation is only casual; given the number of 10^5^ realizations considered, we can reject the null hypothesis, for the significant cases in which no value of *R* is greater or equal to the observed one, with a probability to be wrong lower than 0.00001. In Fig. 2 we show the statistically significant values of *R*, as formerly defined, as squares. We want to highlight this criterion is extremely rigorous (confidence level is very high, 99.999%, with respect to the normally used levels of 95–99%), but in fact our aim is to demonstrate, beyond any reasonable doubt, if correlation between any proton variables and earthquakes does exist. For the same reason we used all the available proton data, even when a single day was preceded and followed by data lacking: this obviously led to *R* value, and hence significance, underestimation.

The analyses so far described, depicted in Fig. 2, show that the condition *1Dy bT* in Table 2 (i.e. one day after the variable decrease below the threshold value) is the only significant one, and only for *ρ* (density) and *ρν* (flux) variables. Moreover it monotonically increases as the threshold value increases, at least up to values of threshold not too high, where the sampling becomes too poor. Such an increasing trend of the R peak value is best observed for the density *ρ*, but it can be observed also, although with lower peak values, for the flux *ρv*. We can therefore state that the most striking correlation between proton variables and global seismicity is with earthquakes occurring during the 1st day after the density value *ρ* decreases below a certain threshold, in the *V*_*step*_ range of 0.31–0.39. Such a range for *V*_*step*_ corresponds to a range of proton density between 12.7 and 15.9 counts cm^−2^.

As a final step, we have further checked the dependence of the observed *R* peak values on the magnitude threshold of the earthquake catalogue. We have then progressively increased the lower magnitude threshold of the used seismic catalogue according to Table 1.

**Figure 4:**
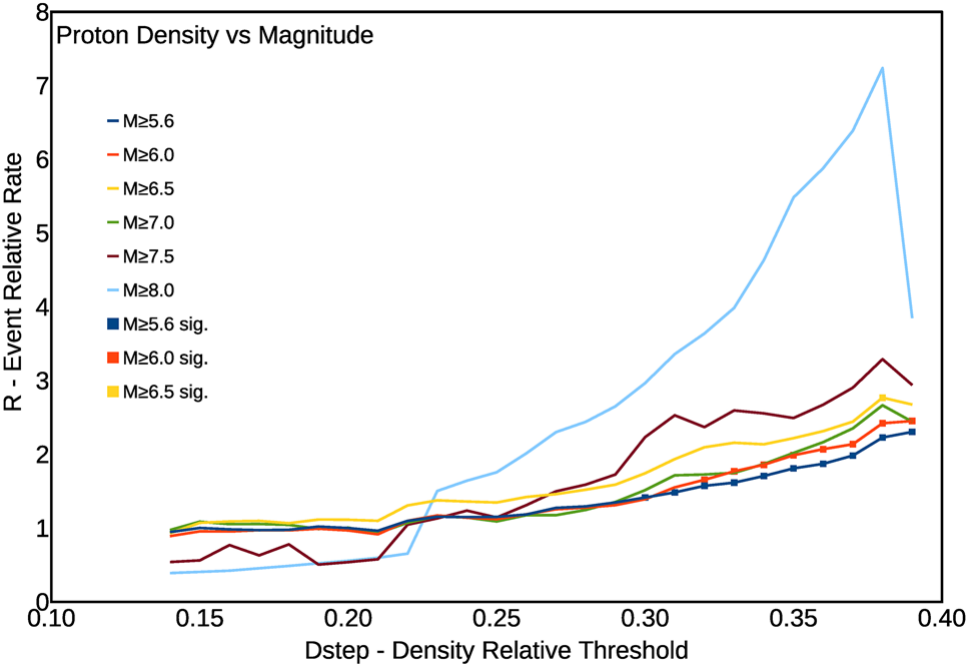
Plots of the Event Relative Rate R as a function of the normalized proton density, and for the condition 1Dy bT (earthquakes occurring within 24 h from the value of density decreasing below the threshold value). Colours indicate different lower cut-off magnitudes in the catalogue.

Figure 4 clearly shows the correlation peak that becomes larger and larger with increasing magnitude cut-off. These results confirm the existence of a strongly significant correlation between worldwide earthquakes and the proton density in the near the magnetosphere, due to solar activity.

### Testing and discussion of statistical results

**Figure 5:**
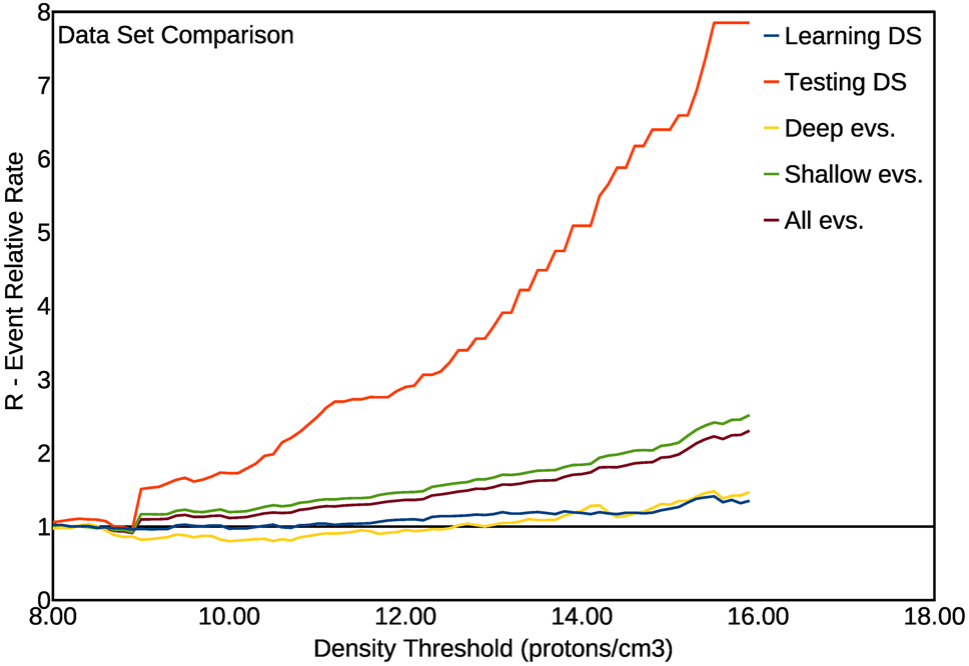
Plots of the Event Relative Rate R as a function of the absolute value of proton density, and for the condition 1Dy bT (earthquakes occurring within 24 h from the value of density decreasing below the threshold value). Colours indicate different subdivisions of the catalogue (the results for the total catalogue are shown by the brown curve).

A further way to check the robustness of the inferred correlation is to divide the catalogue in two parts, using the first half (1996–2005) to infer the best correlation parameters, and then applying the inferred parameters to the second half (2006–2016) to see if it continues to indicate a significant correlation. This is a classical test to verify that the inferred correlation is not just a result of overfitting the data. The first part of the catalogue is the ‘learning’ set, whereas the second part is the ‘testing’ set. If the results obtained with the testing set, using the optimal criteria inferred from the learning one, also indicate a significant correlation, then such a correlation is robust and not just an overfitting of data. We then divided the catalogue in two parts, each one 10 years long as previously indicated, and inferred the best model from the first part. We actually inferred, as the best model (of highest R score) from the first half of the catalogue, the day after the proton density peak decreases below the threshold; then, we computed the R score obtained, with the same model, from the second half of the catalogue. We have then generalized this test, in order to check also the relative performance of the different catalogues containing, respectively, the shallow (Depth < 60 km) and the deep (Depth > 60 km) earthquakes. This further subdivision is also interesting, for two reasons: firstly, the mechanism of deep events could be in principle somewhat different from the crustal ones; secondly (and somewhat related), because they are generally not followed by sustained aftershock sequences. The catalogue of deep events is then a naturally ‘declustered’ one, and so it can help to understand how the proton density–earthquake correlation would work for a declusterd catalogue. Obviously, the process of artificial declustering is a highly subjective one, and could then destroy the main features of the catalogue linked to the correlation^33^. The results obtained for the R score with such different subdivisions of the catalogue are shown in Fig. 5, and compared to the results obtained with the whole catalogue (already shown in Fig. 2a). As it is evident, all the curves, for all the sub-catalogues, show a marked increase of the R score as a function of the proton density threshold used. This makes clear that the effect of increasing seismicity with increasing proton density always occurs. The significance level to accept the observed correlation, computed the same way explained for Fig. 2 (i.e. randomly generating many catalogues sharing the same interevent distribution than the real one) goes from 0.001 of the learning catalogue and catalogue of deep events, to 0.00001 of the whole catalogue, catalogue of shallow events and testing catalogue. We should hence conclude, from this test, that the observed correlation is always significant, that it does not represent an overfitting of data (because the same parameters inferred from the learning catalogue also well describe the testing one), and that also the catalogue of only deep events shows a correlation with proton density. We should also note, however, that the solar activity (proton density) in the second part of the catalogue is significantly lower than in the first part; and such a decrease is particularly marked in the last period (2013–2016) where, for any given density threshold, there are less density peaks and consequently less periods of 24 h following the density peaks.

**Figure 6:**
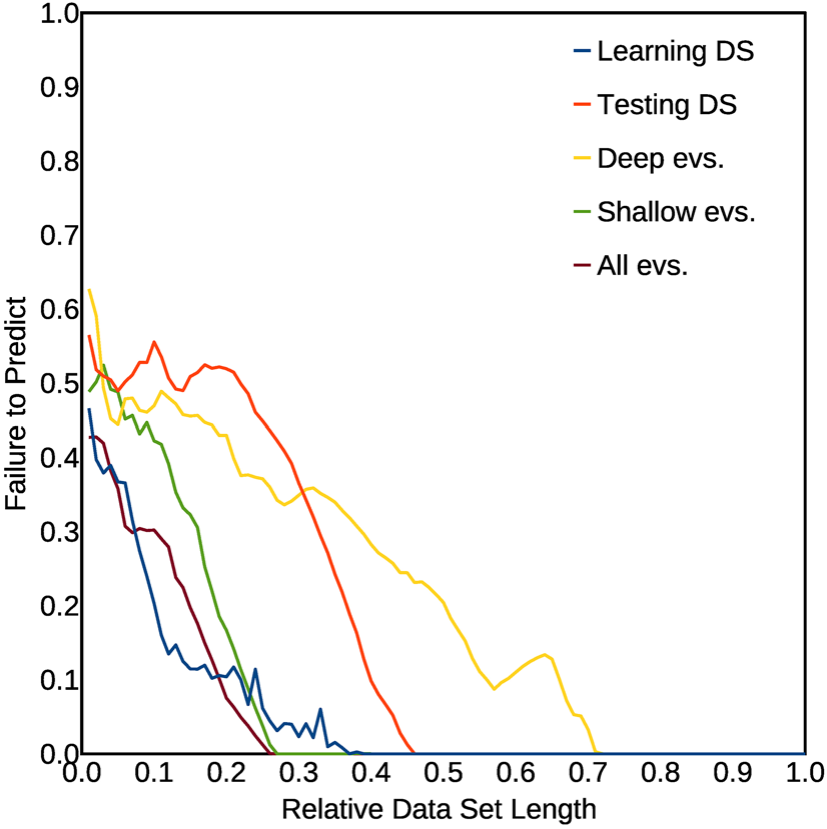
Diagrams showing the fraction of failure to predict as a function of the length of sliding time windows in which the catalogue is subdivided. The length is shown, on the X axis, normalized to the total length of the catalogue. Curves for different catalogues are shown with different colors (see also the text). The results shown here have been computed using the value for the proton density threshold ρ_T_ = 15.5 protons/cm^3^, which represents the optimal value, which minimizes the integral below the curve for the total catalogue. The value of the integral below each curve represents the total prediction failure fraction; it should be close to 0.5 for a random, non predictive model. The values found here are: 0.05 for the total catalogue; 0.05 for the ‘learning’ catalogue; 0.08 for the shallow events catalogue; 0.17 for the ‘testing’ catalogue; 0.21 for the deep events catalogue.

It can be even more interesting to see what happens by subdividing the catalogue in smaller parts, and to study the goodness of the inferred model obtained for different subdivisions, progressively larger, of the catalogue. The procedure here used as a test for the inferred correlation has been adapted from the concept of the Molchan diagram^34^. We started considering a time window whose length in days is 0.01 of the total catalogue: 0.01 × 6,774 = 67.74, rounded to 68 days. We than consider all the parts of length 68 days in the catalogue, by sliding progressively the 68 days windows of 1 day each step, until the 68th day corresponds to the ending day of the catalogue. In this way, we obtain 6,774 − 68 = 6,706 time windows. For each time window, we compute if the Event Relative Rate R, for events occurring within 24 h from the end of a density peak, is higher or lower than the average number of events per day computed from the whole catalogue (0.95 events/day): if it is higher, the prediction outcome is positive, otherwise it is negative. In case there is, in a given time window, no day occurring after the end of a density peak, that window is excluded from the count. On the Y axis, we indicated the fraction of the sliding time windows with a prediction failure. Then, we repeat the same procedure and computation for time windows progressively larger, from a fraction 0.01 to 1 of the total catalogue time duration; for each fractional length of the sliding window, indicated on the X axis, we report on the Y axis the fraction of prediction failures. It is obvious that, for a totally random outcome, the fraction of prediction failures is around 0.5; a number significantly smaller indicates a significant correlation, whereas a number significantly higher would indicate a significant anti-correlation. We have applied this procedure to all the subcatalogues here considered (learning catalogue, testing catalogue, shallow event catalogue, deep event catalogue) as well as to the whole catalogue. Figure 6 shows the results of such analysis, for the best fitting density threshold, computed for the total catalogue, of 15.5 protons/cm^3^. The results synthesized in Fig. 6 are effective to give a complete, clear picture of the robustness of the results, and then of the inferred correlation. In fact, they clearly show values of the failure fraction, for the learning catalogue, the testing catalogue and the total catalogue, which are significantly smaller than 0.5, except for very low time windows. The integral below the respective curves here represent the total failure fraction: it varies from a minimum of 0.05 for the learning catalogue and the whole catalogue, to a maximum of 0.21 for the catalogue of deep earthquakes. It is also worthy of note that some catalogues show even better results with slightly different density thresholds. The catalogue of deep earthquakes, for instance, has a total failure fraction of only 0.14 for a density threshold slightly higher: 16.1 protons/cm^3^; the ‘testing’ catalogue has a better minimum of total failure fraction of 0.14 for a lower density threshold: 13.3 protons/cm^3^. So, all the obtained values of global failure fraction are considerably smaller than 0.5, thus confirming the predictivity of the method, and then the significance of the correlation. We should note that the method we use here conceptually differs from the Molchan diagram also for the shape of the space involved to discriminate purely randomly results from a significant correlation. In fact, a purely random result is represented as the diagonal of a square with surface normalized to 1.0 in the Molchan diagram^34^; in our method, conversely, a purely random result is represented by a horizontal line with a constant value Y = 0.5. In both diagrams, the surface (integral) below a curve representing purely random results has a value around 0.5; significantly lower values indicate, on the contrary, significant degree of predictivity.

All the obtained results and tests point out the correlation between earthquakes and proton density is highly statistically significant, even if for catalogues with too large earthquake magnitude thresholds it does not strictly pass the significance test. This is due to the fact that the three higher magnitude data sets (*M* ≥ 7.0, 7.5, 8.0) are composed by a really small number of events (Table 1) and furthermore, for such reason, the Gutenberg–Richter relation is no longer valid.

**Figure 7:**
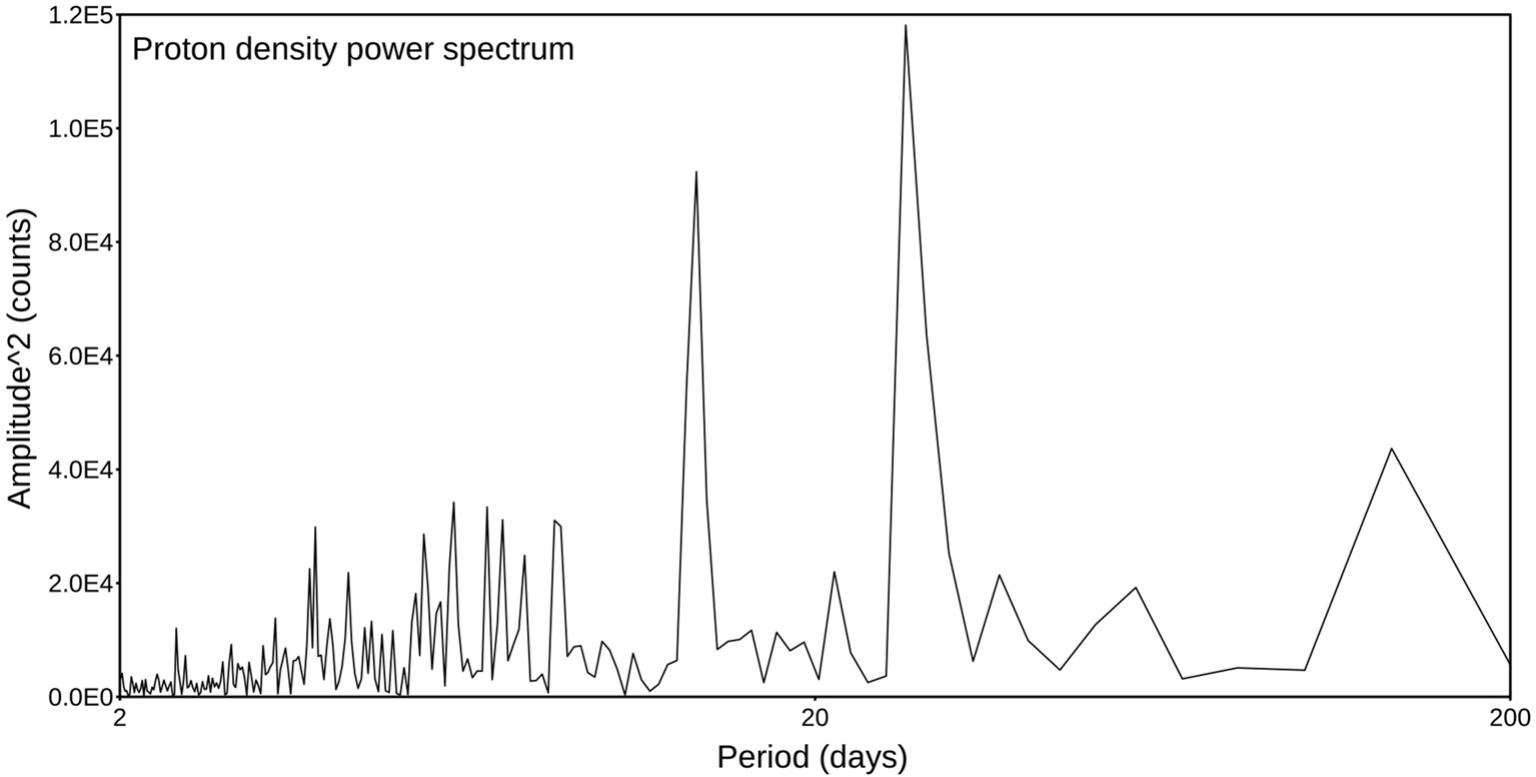
Power spectrum computed for the proton density catalogue. The spectrum is computed only for the maximum consecutive period of data with no interruption, lasting 200 days.

As a final test, we wanted to check if the proton density catalogue is completely uncorrelated. We know, as stated above, that the seismic catalogue of strong earthquakes is non-Poissonian and internally correlated, so we have analyzed the proton density series to check if it were characterized by a white noise spectrum that would indicate an uncorrelated process. We simply computed the power spectrum, which is shown in Fig. 7; it is clearly very different from a white spectrum, presenting at least two sharp peaks. We performed such a computation on the longest uninterrupted time window that has a 405 days length. This evidence testifies that neither the proton density distribution is random. So, this definitively confirms that the observed correlation between the seismic catalogue and the proton density cannot be likely obtained by chance; because the likelihood that two quantities, each of them internally correlated, show a clear mutual correlation only by chance is negligible.

In conclusion, the analysis of the 1996–2016 worldwide earthquake catalogue shows a significant correlation with the measured proton density in the same period. Such correlation is described by a larger probability for earthquakes to occur during time windows 24 h long just after a peak period (meant as a period spent over a certain threshold) in proton density due to solar activity. This kind of correlation between worldwide seismicity and solar activity has been checked also with other variables linked to solar activity, including proton velocity, dynamical pressure of protons, proton flux, and proton density. However, a significant correlation can be only observed with proton flux, besides proton density. The correlation is anyway much sharper using simple proton density, so evidencing that this is the really influent variable to determine correlation with earthquake occurrence. This correlation is shown to be statistically highly significant. The high significance of the observed correlation is also strengthened by the observation that, increasing the threshold magnitude of the earthquake catalogue, the correlation peak becomes progressively larger. The application of a further appropriate methodology of testing, using concepts similar to the Molchan diagram^34,35^, also confirms the statistical significance of the observed correlation. The correlation between large earthquakes worldwide and proton density modulated by solar activity then appears to be strongly evident and significant.

### A possible qualitative model to explain observations

Once a strong correlation between proton density, generated by solar wind, and large earthquakes worldwide has been assessed, the next step is to verify if a physical mechanism exists which could explain such a result. Several mechanisms have been proposed, till now, for solar-terrestrial triggering of earthquakes (see^26^ for a review). Although former observations about solar-terrestrial triggering were not convincing^26^, some of the formerly proposed mechanisms could explain our results, which are on the contrary statistically significant. In particular, Sobolev and Demin^25^ studied the piezoelectric effects in rocks generated by large electric currents. Our observed correlation implies that a high electric potential sometimes occurs between the ionosphere, charged by the high proton density generated at higher distances, and the Earth. Such a high potential could generate, both in a direct way or determining, by electrical induction, alterations of the normal underground potential, an electrical discharge, channeled at depth by large faults, which represent preferential, highly conductive channels. Such electrical current, passing through the fault, would generate, by reverse piezoelectric effect, a strain/stress pulse, which, added to the fault loading and changing the total Coulomb stress, could destabilize the fault favoring its rupture. The reverse piezoelectric effect would be due, in rocks, by the quartz minerals abundant in them. Such effect can work, in principle, for all kinds of faults. The piezoelectric effect, in fact, acts to produce a pulse of dilatation or contraction on a particular axis of the crystal, depending on the polarity of the electrical current. For quartz crystals randomly distributed on a fault surface of any orientation, the net effect is a pulse of strain/stress normal to the fault, because the other strain/stress components compensate among them into the bulk rocks. The normal stress can stabilize or destabilize any kind of fault, depending on the sign^36–38^; however, since it is a transient pulse, it has an effect only in case it is able to instantaneously increase the total Coulomb stress on a given fault above the fracture strength, thus generating the earthquake^36^. It would then represent only a small destabilizing effect over an already critically loaded fault. So, the earthquake cycle would be anyway dominated by tectonic phenomena, but this small external triggering effect could generate the observed slight correlation among worldwide earthquakes. These kinds of effects, induced by high electrical potential between the ionosphere and the Earth, should likely be accompanied by electrical discharges in atmosphere, which would cause luminescence phenomena. Actually, there are numerous observations of macroscopic luminescence phenomena (named Earthquake Lights) before and accompanying large earthquakes^39^. Moreover, these phenomena could also cause strong electromagnetic effects, which would be recorded as radio-waves; even such phenomena have been largely reported as accompanying, and generally preceding, large earthquakes^40^. More in general, a lot of electro-magnetic anomalies, often well evident, are more and more frequently reported associated to moderate to large earthquakes^41^. The recent scientific literature is full of hypotheses about how such electromagnetic effects, associated to large earthquakes, could be generated. The most debated question is if they can be considered as precursors (or maybe triggers) for large events, or they are caused by the process of slip on the faults which also generate the earthquake^42,43^. Here we suggest that the increase in the proton density near the magnetosphere can qualitatively explain all these observations, and also give a physical basis to our statistical observations.

## Conclusions

This paper gives the first, strongly statistically significant, evidence for a high correlation between large worldwide earthquakes and the proton density near the magnetosphere, due to the solar wind. This result is extremely important for seismological research and for possible future implications on earthquake forecast. In fact, although the non-poissonian character, and hence the correlation among large scale, worldwide earthquakes was known since several decades, this could be in principle explained by several mechanisms. In this paper, we demonstrate that it can likely be due to the effect of solar wind, modulating the proton density and hence the electrical potential between the ionosphere and the Earth. Although a quantitative analysis of a particular, specific model for our observations is beyond the scope of this paper, we believe that a possible, likely physical mechanism explaining our statistical observations, is the stress/strain pulse caused by reverse piezoelectric effects. Such pulses would be generated by large electrical discharges channeled in the large faults, due to their high conductivity because of fractured and water saturated fault gauge. The widespread observations of several macroscopic electro-magnetic effects before, or however associated to large earthquakes, support our qualitative model to explain the observed, highly statistically significant, proton density-earthquakes correlation. It is important to note that our hypothesis only implies that the proton density would act as a further, small trigger to cause the fracture on already critically charged faults, thus producing the observed large scale earthquake correlation. Such a small perturbation would add to the main factor producing worldwide seismicity, which is tectonic stress.
